# Changes in bone marrow morphology in adults receiving romiplostim for the treatment of thrombocytopenia associated with primary immune thrombocytopenia

**DOI:** 10.1007/s00277-016-2682-2

**Published:** 2016-04-30

**Authors:** Ann Janssens, Francesco Rodeghiero, David Anderson, Beng H. Chong, Zoltán Boda, Ingrid Pabinger, Libor Červinek, Deirdra R. Terrell, Xuena Wang, Janet Franklin

**Affiliations:** Department of Hematology, University Hospitals Leuven, Campus Gasthuisberg, Leuven, Belgium; Department of Cell Therapy and Hematology, San Bortolo Hospital, Viale Rodolfi, 37, 36100 Vicenza, Italy; Dalhousie University, QEII—Bethune Building, Suite 442 Bethune Building, 1276 South Park Street, Halifax, NS B3H 2Y9 Canada; St. George Hospital, UNSW Medicine, UNSW, Sydney, 2052 Australia; Clinical Center Department of Medicine, Thrombosis Haemostasis Center, University of Debrecen, H-4012 Debrecen, Nagyerdei krt. 98. POB.: 20, Debrecen, Hungary; Medizinische Universitaet Wien, Universitaetsklinik fuer Innere Medizin I Waehringer Guertel 18-20, A-1090 Vienna, Austria; University Hospital Masaryk University, Jihlavská 340/20, 625 00 Brno, Czech Republic; Biostatistics and Epidemiology, University of Oklahoma Health Sciences Center, 801 NE 13th, CHB 333, Oklahoma City, OK 73104 USA; Amgen Inc., One Amgen Center Drive, Thousand Oaks, CA 91320 USA

**Keywords:** Immune thrombocytopenia (ITP), Thrombopoietin (TPO), Platelet, Bone marrow, Reticulin, Collagen

## Abstract

The effects of romiplostim on bone marrow morphology were evaluated in adults with immune thrombocytopenia (ITP). Patients with platelet counts <50 × 10^9^/L, ≥1 prior ITP therapies, and no collagen at baseline received weekly subcutaneous romiplostim starting at 1 μg/kg, adjusted to maintain platelet counts between 50 and 200 × 10^9^/L. Biopsies were scheduled after 1, 2, or 3 years of romiplostim (cohorts 1, 2, and 3, respectively). Irrespective of scheduled time, biopsies were performed earlier if patients discontinued or failed to achieve/maintain a response to romiplostim. Reticulin (silver stain) and collagen (trichrome stain) were graded by two hematopathologists using the modified Bauermeister scale (0–4). Of 169 patients, 131 had evaluable biopsies; 9/131 (6.9 %) had increases of ≥2 grades on the modified Bauermeister scale (cohort 1: 0/34; cohort 2: 2/39; cohort 3: 7/58), including two with collagen. Three of the nine patients had follow-up biopsies, including one patient with collagen; changes were reversible after romiplostim discontinuation. Of the nine patients, one had neutropenia detected by laboratory test and two had adverse events of anemia, both non-serious and not treatment-related. By actual exposure (as some biopsies did not occur as scheduled), the number of patients with grade increases ≥2 were year 1: 3/41, year 2: 1/38, year 3: 5/52. Twenty-four patients sustained platelet counts ≥50 × 10^9^/L for ≥6 months with no ITP medications after discontinuing romiplostim, i.e., they entered clinical remission of their ITP. In conclusion, in patients with ITP receiving romiplostim, bone marrow changes were observed in a small proportion of patients.

ClinicalTrials.gov identifier: NCT#00907478

**Electronic supplementary material:**

The online version of this article (doi:10.1007/s00277-016-2682-2) contains supplementary material, which is available to authorized users.

## Introduction

Primary immune thrombocytopenia (ITP) is an autoimmune disorder characterized by low platelet counts caused by both increased platelet destruction and insufficient platelet production [1]. Chronic ITP typically lasts longer than 12 months and rarely remits spontaneously [1]. The disorder commonly manifests in the form of epistaxis, gingival bleeding, petechiae, and bruising. Less commonly, more severe events such as gastrointestinal bleeding and, in rare cases, intracranial bleeding can occur [2].

First-line therapy for ITP includes corticosteroids, intravenous immunoglobulin (IVIg), and anti-D immunoglobulin [3–5]; however, the efficacy and durability of response for these and subsequent treatments, such as splenectomy, azathioprine, vincristine, and rituximab, vary greatly and can be associated with toxicities that limit their extended use [2, 4–6]. Romiplostim, a thrombopoietin (TPO) receptor agonist that activates intracellular transcriptional pathways to stimulate megakaryopoiesis and increase platelet production [7], can increase platelet counts and reduce bleeding and the use of concomitant ITP medications in patients with newly diagnosed, persistent, and chronic ITP [8–11].

Reticulin is a normal component of the bone marrow stroma; increased reticulin is associated with various benign conditions (e.g., treatment with hematopoietic growth factors), whereas collagen fibrosis is a characteristic of myeloproliferative disease [3]. Several different grading methods can be used to quantify reticulin [12]. In bone marrow biopsies analyzed using the modified Bauermeister scale, 95 % of healthy individuals had grade 0–1 bone marrow reticulin and 5 % had grade ≥2 bone marrow reticulin [12]. A retrospective analysis of bone marrow biopsies from 40 patients with ITP reported that reticulin was present in approximately two thirds of patients, with 50 % having grade 1, 2 % grade 0–1, 13 % grade 1–2, and 2 % grade 2 reticulin [13]. Another study found that one third of patients with ITP had increased reticulin (grade 1–2) in the bone marrow; no correlation was found between the presence of reticulin and disease severity or other clinical measures [14].

In thrombocytopenic patients with ITP, treatment with TPO receptor agonists may be associated with increases in bone marrow reticulin [8, 15–20]. The primary objective of this study was to prospectively evaluate bone marrow biopsies for collagen before and after treatment with romiplostim in adult patients with ITP; secondary objectives included assessment of reticulin as well as safety.

## Methods

**Fig. 1:**
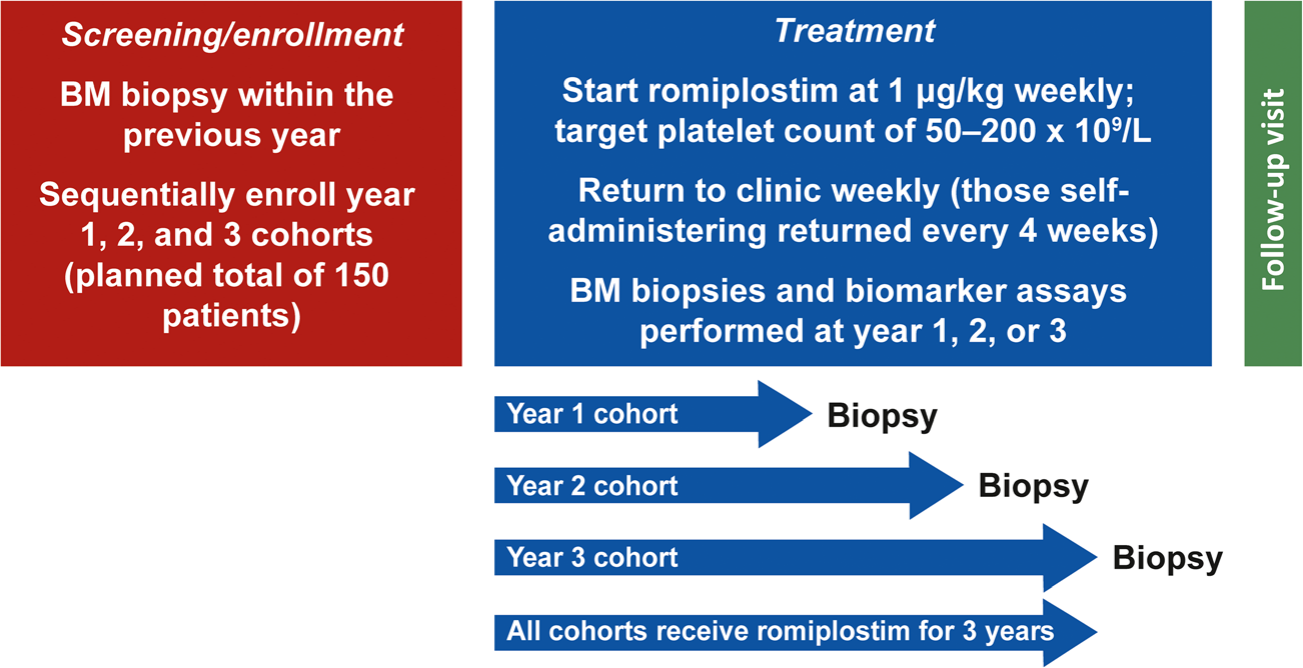
Design of a study to measure changes in bone marrow morphology in patients with ITP receiving romiplostim. *BM* bone marrow, *ITP* immune thrombocytopenia

This phase 4, prospective, open-label, multicenter study evaluated changes in bone marrow reticulin and collagen in patients with ITP receiving romiplostim (Fig. 1). The study was approved by the relevant institutional review boards or ethics committees and conducted in accordance with the Helsinki Declaration. All patients gave written informed consent. Eligible patients were ≥18 years of age with ITP per American Society of Hematology guidelines and had platelet counts <50 × 10^9^/L, ≥1 prior ITP therapy (e.g., corticosteroids, IVIg, or splenectomy), and a baseline bone marrow biopsy negative for collagen.

The primary endpoint was the incidence of collagen in biopsies after up to 1, 2, or 3 years of romiplostim treatment (cohorts 1, 2, and 3, respectively). Biopsies were performed earlier if patients discontinued early or failed to achieve/maintain a response to romiplostim (platelet counts ≤20 × 10^9^/L for 4 consecutive weeks at the maximum romiplostim dose). Patients from cohorts 1 and 2 could continue to receive romiplostim after biopsy for up to a total of 3 years. Reticulin and collagen were measured using the modified Bauermeister scale (Online Resource Table S1) [12]. Grades 0–3 measure changes in reticulin (silver staining), whereas grade 4 is defined as a diffuse, often coarse reticulin fiber network with areas of collagenization (trichrome staining) [12]. All samples were read by two hematopathologists at a central laboratory, with grading discrepancies adjudicated by an independent bone marrow panel. Secondary endpoints included the incidence of bone marrow reticulin increases of ≥2 severity grades over baseline or to grade 4 (collagen).

Safety endpoints included adverse events (AEs), the incidence of neutralizing antibody formation to romiplostim or cross-reacting antibody to endogenous TPO, and the incidence of Common Terminology Criteria for Adverse Events grade ≥2 shift in white blood cell (neutropenia) or red blood cell (anemia) AEs or laboratory values. AEs were specifically monitored for myelofibrosis, defined as proliferation of abnormal bone marrow stem cells resulting in fibrosis or the replacement of the marrow with collagenous connective tissue fibers.

Patients received weekly subcutaneous romiplostim injections for up to 3 years, starting at 1 μg/kg, with weekly adjustment in increments of 1 μg/kg up to a maximum of 10 μg/kg to target platelet counts between 50 and 200 × 10^9^/L. Patients also received any prescribed concomitant ITP medications or treatments deemed necessary to provide adequate supportive care (e.g., corticosteroids). Romiplostim was initially administered at clinic visits. At the investigator’s discretion, patients who achieved a platelet count ≥50 × 10^9^/L without dose adjustments for 4 consecutive weeks were eligible to self-administer romiplostim or have the injection administered by a caregiver. Patients did not necessarily self-administer romiplostim continuously. A post hoc analysis was performed to identify patients exhibiting clinical remission, i.e., sustained platelet counts ≥50 × 10^9^/L for ≥6 months with no ITP medications after discontinuing romiplostim.

The analysis of study endpoints included all patients who received at least one dose of romiplostim. All endpoints were summarized descriptively; continuous endpoints were summarized by mean (standard deviation), median (quartile 1 [Q1], quartile 3 [Q3]), minimum, and maximum values; categorical variables were summarized by frequency or percentage in each category.

## Results

### Enrollment, disposition, and exposure

At baseline, overall, patients (*n* = 169) had a mean (standard deviation) age of 50 (17) years; 68 % were women, median baseline platelet count was 23 × 10^9^/L (range, 0.5–130), and 60 (36 %) patients had a splenectomy before enrollment (Table 1). Cohorts were sequentially enrolled—50 in cohort 1 (bone marrow biopsy planned for after 1 year of romiplostim), 50 in cohort 2 (biopsy planned after 2 years), and 69 in cohort 3 (biopsy planned after 3 years). Patients in cohorts 1 and 2 had the option of continuing to receive romiplostim past the time of biopsy to a maximum total of 3 years. A total of 103 (61 %) patients completed all 3 years of the study (Online Resource Table S2). The median (Q1, Q3) duration of treatment was cohort 1, 147 (17, 156) weeks; cohort 2, 155 (66, 156) weeks; and cohort 3, 155 (66, 156) weeks.

**Table 1:** Baseline characteristics

Characteristic	Cohort 1 (*N* = 50)	Cohort 2 (*N* = 50)	Cohort 3 (*N* = 69)	All (*N* = 169)
Female, *n* (%)	27 (54.0)	38 (76.0)	49 (71.0)	114 (67.5)
Age, mean (SD), years	55.5 (17.1)	48.6 (16.5)	46.6 (16.3)	49.8 (16.9)
Platelet count^a^, median (range), ×10^9^/L	26 (0.51–30.0)	18 (1.0–93.0)	25 (1.0–74.0)	23 (0.5–130.0)
Duration of ITP, median (range), years	7 (0–48)	5 (0–46)	2 (0–31)	4 (0–48)
Four or more prior ITP treatments, *n* (%)	15 (30)	10 (20)	10 (15)	35 (21)
Splenectomy prior to study, *n* (%)	22 (44)	15 (30)	23 (33)	60 (36)
Bone marrow reticulin and/or collagen grade per modified Bauermeister scale^b^, *n* (%)				
0	12 (24)	15 (30)	16 (23)	43 (25)
1	37 (74)	32 (64)	50 (73)	119 (71)
2	1 (2)	3 (6)	3 (4)	7 (4)

Per study design, patients were sequentially enrolled into three cohorts, with biopsies to be done at baseline and year 1 (cohort 1), year 2 (cohort 2), or year 3 (cohort 3). Patients from cohorts 1 and 2 had the option to continue receiving romiplostim after bone marrow biopsies for a total of 3 years

*ITP* immune thrombocytopenia, *SD* standard deviation

^a^Defined as the last platelet count prior to the first dose of romiplostim

^b^There were no patients with grade 3 or grade 4 biopsies at baseline. Per protocol, the maximum allowable baseline grade was 3

### Bone marrow and efficacy

**Fig. 2:**
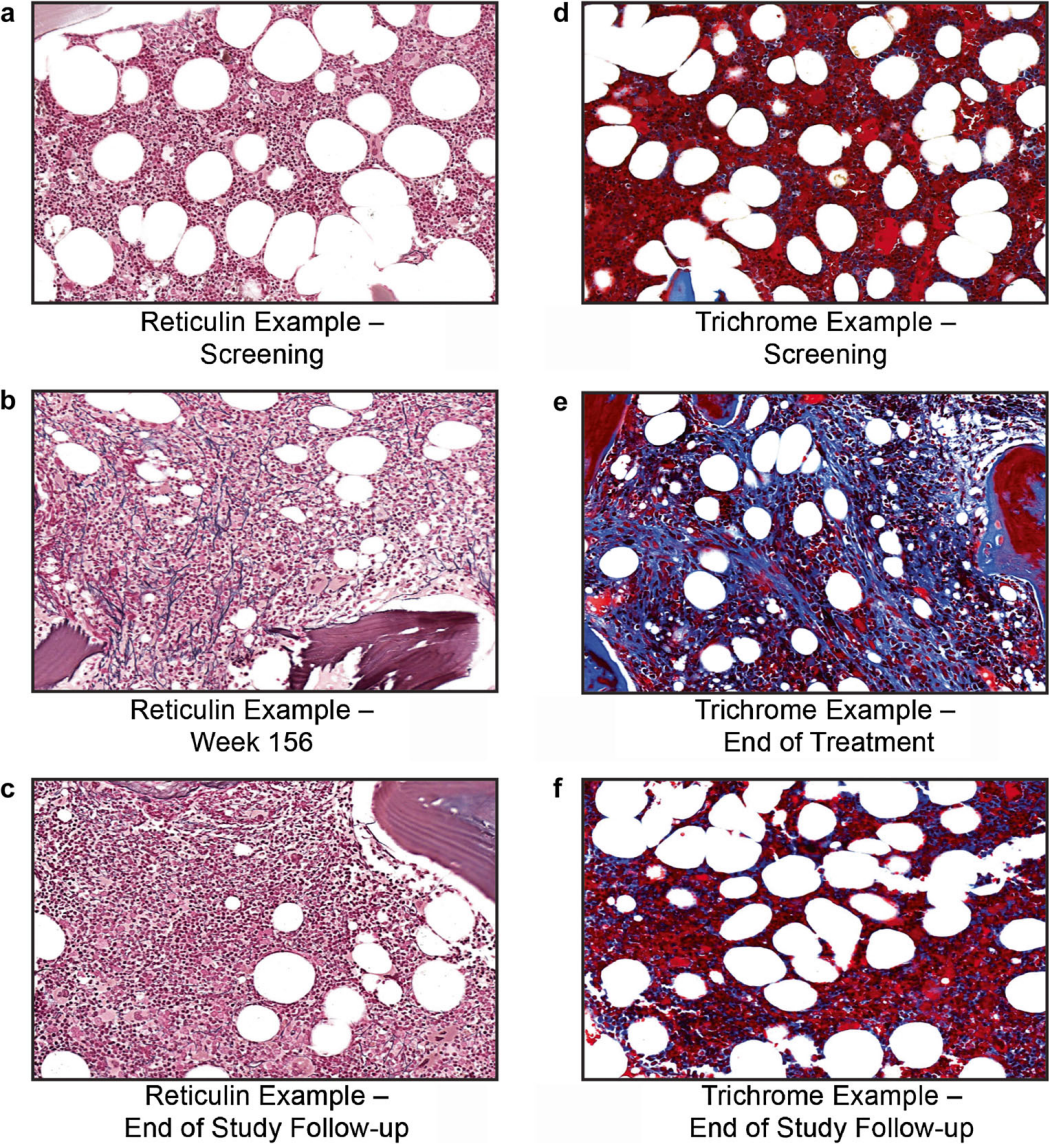
Images from bone marrow biopsies of patients with ITP treated with romiplostim. Bone marrow images of a patient with reversible increased reticulin (silver stain) (**a**–**c**) and of another patient with reversible collagen (trichrome stain) (**d**–**f**). *Images* are arranged as follows: screening (**a**, **d**), end of treatment (**b**, **e**), and follow-up after discontinuation (**c**, **f**). As not all biopsies were stained at the same time; there were slight differences in staining intensity, brightness, and tone between timepoints for a single patient. To more easily allow for comparison of images of a single patient and to correct for scanning defects, brightness, contrast, and saturation were adjusted using PowerPoint. *ITP* immune thrombocytopenia

Of the patients with evaluable biopsies, two patients in cohort 2 and five in cohort 3 had an increase in reticulin by ≥2 grades (not including collagen) on the modified Bauermeister scale; two of the patients in cohort 3 had collagen (top half of Table 2, patient details in Table 3). Three of the nine patients with bone marrow changes had follow-up biopsies after romiplostim discontinuation. Of these, two patients with an on-study increase from grade 1 to grade 3 returned to grade 1 after 12 weeks (bone marrow biopsy images from one of these patients are shown in Fig. 2a–c). For the third patient, collagen was observed after 25 weeks of romiplostim treatment; there was no evidence of collagen (grade 2 reticulin only) at follow-up for 10 weeks after romiplostim discontinuation (bone marrow biopsy images are shown in Fig. 2d–f). The bone marrow biopsy on this patient with collagen was performed early due to prolonged non-response at the maximal dose of romiplostim (10 μg/kg). This patient had ITP that was unresponsive to treatment with multiple corticosteroids, danazol, IVIg, and cyclosporine. Per patient refusal, there was no follow-up biopsy for the other patient with collagen, which was found at the scheduled 3-year biopsy. Three of the nine patients with bone marrow changes discontinued treatment early: (1) one due to requirement for alternative therapy (discontinued at week 10)—this patient later had a splenectomy; (2) the aforementioned case with collagen discontinued after 25 weeks; and (3) one due to work-up to rule out non-Hodgkin lymphoma (discontinued at week 13; suspicion was due to atypical lymphocytes in the peripheral blood, and biopsy after treatment contained grade 3 reticulin)—the patient was judged as not having non-Hodgkin lymphoma. As noted above, biopsies did not always occur as scheduled, such as when patients discontinued early; results by actual exposure duration at time of biopsy are in the bottom half of Table 2. Grade changes in the modified Bauermeister grading scale for all patients are shown in Online Resource Table S3.

**Table 2:** Number of patients with reticulin or collagen present in bone marrow biopsies as determined by silver or trichrome staining and the modified Bauermeister scale

By cohort	Cohort 1 (*N* = 50)	Cohort 2 (*N* = 50)	Cohort 3 (*N* = 69)	Total (*N* = 169)
Bone marrow biopsies after receiving romiplostim^a^	39	40	58	137
Biopsies evaluable for collagen (trichrome stain)^b^	35	39	58	132
Positive for collagen	0	0	2 (3.4 %)^c^	2 (1.5 %)^c^
Patients with biopsies evaluable for reticulin (silver stain)^b^	34	39	58	131
Increase in reticulin by at least two grades excluding collagen	0	2 (5.1 %)	5 (8.6 %)^d^	7 (5.3 %)
By exposure at time of biopsy	1 year^e^	2 years^e^	3 years^e^	Total (*N* = 169)
Biopsies evaluable for collagen (trichrome stain)^b^	42	38	52	132
Positive for collagen	1 (2.4 %)	0	1 (1.9 %)	2 (1.5 %)^c^
Patients with biopsies evaluable for reticulin (silver stain)^b^	41	38	52	131
Increase in reticulin by at least two grades excluding collagen	2 (4.9 %)	1 (2.6 %)	4 (7.7 %)	7 (5.3 %)

*ITP* immune thrombocytopenia

^a^Three patients in cohort 1, three in cohort 2, and 10 in cohort 3 had biopsies at the end of treatment because of early discontinuation. Reasons included lack of response/requirement for alternative therapy (*n* = 9), consent withdrawn (*n* = 4), suspected non-Hodgkin lymphoma (*n* = 1), difficulty dosing (*n* = 1), and patient ineligibility (*n* = 1)

^b^Trichrome and silver staining results could not be obtained for all biopsies because of inadequate samples. Per bone marrow biopsy (smear and core) comments, these biopsies had insufficient cellular marrow for evaluation

^c^One case: grade 4 after 25 weeks in a patient with ITP who did not respond to multiple courses of treatment; no evidence of collagen on follow-up biopsy 10 weeks later. One case: grade 4 at end of treatment; patient refused follow-up biopsy

^d^The five patients with increases in reticulin by two or more grades (excluding those patients with collagen) included two patients who increased from grade 0 to 2 and three patients who increased from grade 1 to 3

^e^Year 1 was defined as up to 1.5 years as some patients did not have their scheduled biopsy until months later; likewise for cohorts 2 and 3

**Table 3:** Characteristics of patients with changes in bone marrow of two or more grades on the modified Bauermeister scale

Cohort	Age, years	Sex	Race	Prior splenectomy	Time with platelet response, %	Median (Q1, Q3) romiplostim dose, μg/kg	Total weeks on drug	Collagen	Cytopenia
2	49	M	White	N	82	3 (3, 3)	156^**a**^		
	48	F	White	Y	0	7 (4, 10)	13		
3	81	M	White	N	0	9 (8, 9)	156		Anemia
	30	M	White	N	0	10 (7, 10)	26	Present	
	32	F	White	N	72	10 (7, 10)	156		
	60	M	White	Y	5	10 (10, 10)	156	Present	
	29	F	White	N	72	9 (5, 10)	156		Neutropenia
	47	F	White	Y	84	9 (9, 9)	156		
	53	F	White	N	0	5 (2, 7)	9		Anemia

Cytopenias were defined as Common Terminology Criteria for Adverse Events grade 2 or greater shift in white blood cell (neutropenia) or red blood cell (anemia) adverse events or laboratory values

*F* female, *M* male, *N* no, *Q1* quartile 1, *Q3* quartile 3, *Y* yes

^a^As patients were allowed to remain on romiplostim for 3 years (i.e., even after biopsy for patients in cohorts 1 and 2), this patient had the option of continuing treatment, which he did for another 52 weeks after biopsy at week 104

**Fig. 3:**
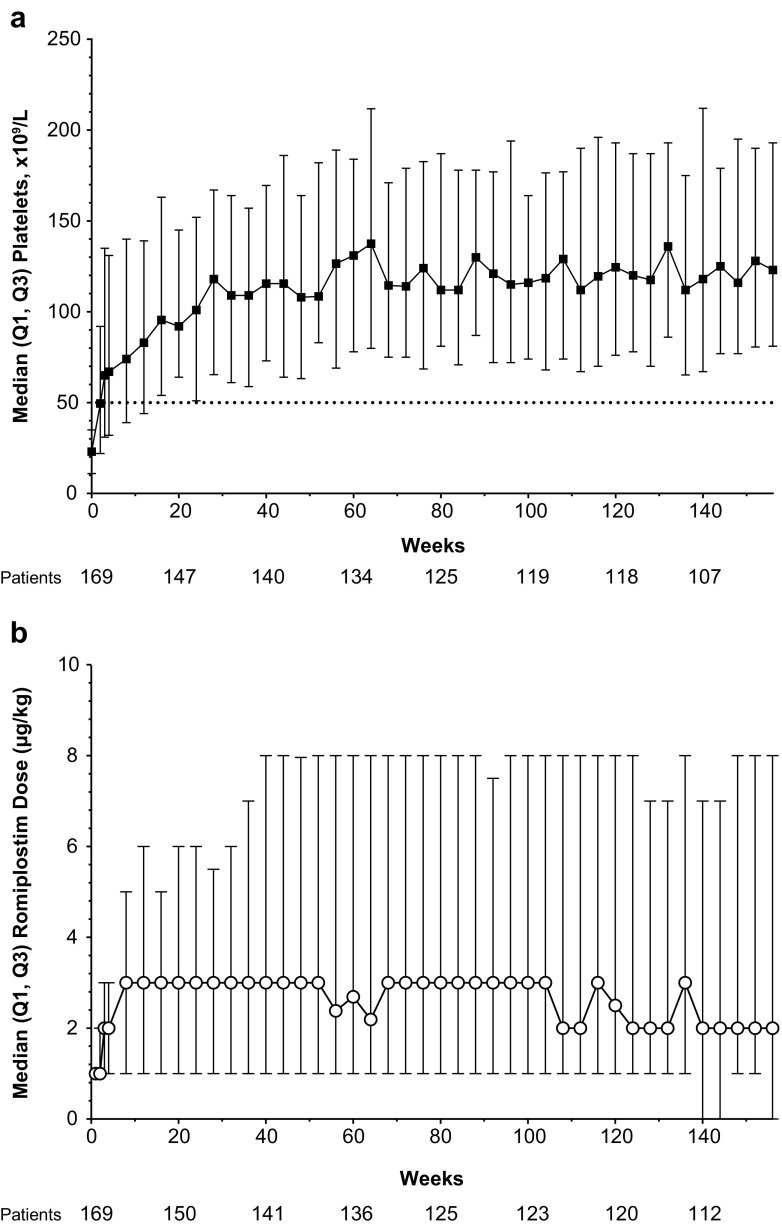
Platelet count and dose over time for patients with ITP treated with romiplostim. **a** Platelet count over time. A platelet count of ≥50 × 10^9^/L was achieved by 25 % of patients after 1 week and by 50 % of patients after 2 weeks. **b** Romiplostim dose over time. *ITP* immune thrombocytopenia, *Q1* quartile 1, *Q3* quartile 3

Platelet count and romiplostim dose over time for all patients are shown in Fig. 3. Platelet counts of ≥50 × 10^9^/L were achieved by 25 % of patients after week 1 and by 50 % of patients after week 2. The median romiplostim dose for the overall population was relatively stable over time. During the course of the study, 24 patients (14 %) entered clinical remission, i.e., they sustained platelet counts ≥50 × 10^9^/L for ≥6 months with no ITP medications after discontinuing romiplostim. None of these patients had bone marrow changes of increased reticulin of ≥2 grades or collagen. Of the 24 patients with remission, three were from cohort 1, seven from cohort 2, and 14 from cohort 3. Median time of onset for observed remission was 52 weeks (range, 6–124 weeks), and median duration of remission observed during the study was 88 weeks (range, 29–154 weeks); 21 of the 24 patients were still in remission at last observation. There were no notable differences between those who did and did not enter remission in baseline demographics, disease characteristics, and other measures (such as baseline platelet count, time to first platelet response, and average weekly dose).

### Adverse events

Online Resource Table S4 shows incidences of AEs for each cohort including all time on romiplostim, i.e., including time past biopsy for those in cohorts 1 and 2 who chose to continue treatment. Seven deaths occurred during the study; none were attributed to romiplostim (Online Resource Table S5). Treatment-related serious AEs were seen in six patients: pulmonary embolism in two patients and exostosis, pulmonary thrombosis, lichenoid keratosis, and peripheral embolism in one patient each (i.e., separate AEs). Twenty-one thromboembolic AEs occurred in 15 (9 %) patients at a rate of 3.9 per 100 patient-years; events reported in more than one patient included deep vein thrombosis (*n* = 4) as well as cerebrovascular accident, pulmonary embolism, thrombophlebitis superficial, and thrombosis (all *n* = 2). Twelve thromboembolic AEs occurring in 11 patients were considered serious, including two instances each of cerebrovascular accident, pulmonary embolism, and deep vein thrombosis and one instance each of portal vein thrombosis, pulmonary thrombosis, ischemic stroke, peripheral embolism, myocardial infarction, and thrombosis, the latter two occurring in the same patient. Platelet counts prior to the thromboembolic AEs ranged from 15 × 10^9^/L to 333 × 10^9^/L, with a median of 157 × 10^9^/L and 7/21 being ≥200 × 10^9^/L. Of the 21 instances of thromboembolic AEs, in 11 cases romiplostim was continued as before; in seven cases the dose was withheld or altered; and in three cases romiplostim was discontinued. One patient in cohort 3 tested positive for neutralizing antibodies to romiplostim at week 30 through the year 1 visit but was negative on a subsequent test (week 72); the last romiplostim dose was at week 36, but the patient maintained platelet response throughout. No patient developed neutralizing antibodies to TPO.

Cytopenias occurred in three (33 %) patients with bone marrow changes (Table 3) and in 17 (14 %) patients without bone marrow changes (6 with anemia, 12 with neutropenia, 1 with both); of note, these cytopenias included changes in laboratory values which were not necessarily of clinical significance (i.e., no symptoms or other findings). In the three patients with bone marrow changes and cytopenias (all ≥2 grade reticulin, none collagen), two had anemia and one had neutropenia; none of the nine patients with bone marrow changes had both anemia and neutropenia. The case of grade 2 neutropenia was detected by a change in laboratory values only; there was no associated AE. Both cases of anemia were reported as AEs; neither was considered serious or treatment-related by the treating physicians. One patient had intraoperative bleeding during a splenectomy and the second had a history of esophageal and duodenal ulcers and current gastrointestinal bleeding. The small number of patients with bone marrow changes and cytopenias precluded any statistical comparisons. AEs were also examined by reticulin status (Online Resource Table S6). While the data do not support a formal statistical comparison, the data suggest that there were no notable differences in AEs (i.e., in proportion of AEs by grade, leading to discontinuation, etc.) between those with and without bone marrow changes.

### Self-administration

**Fig. 4:**
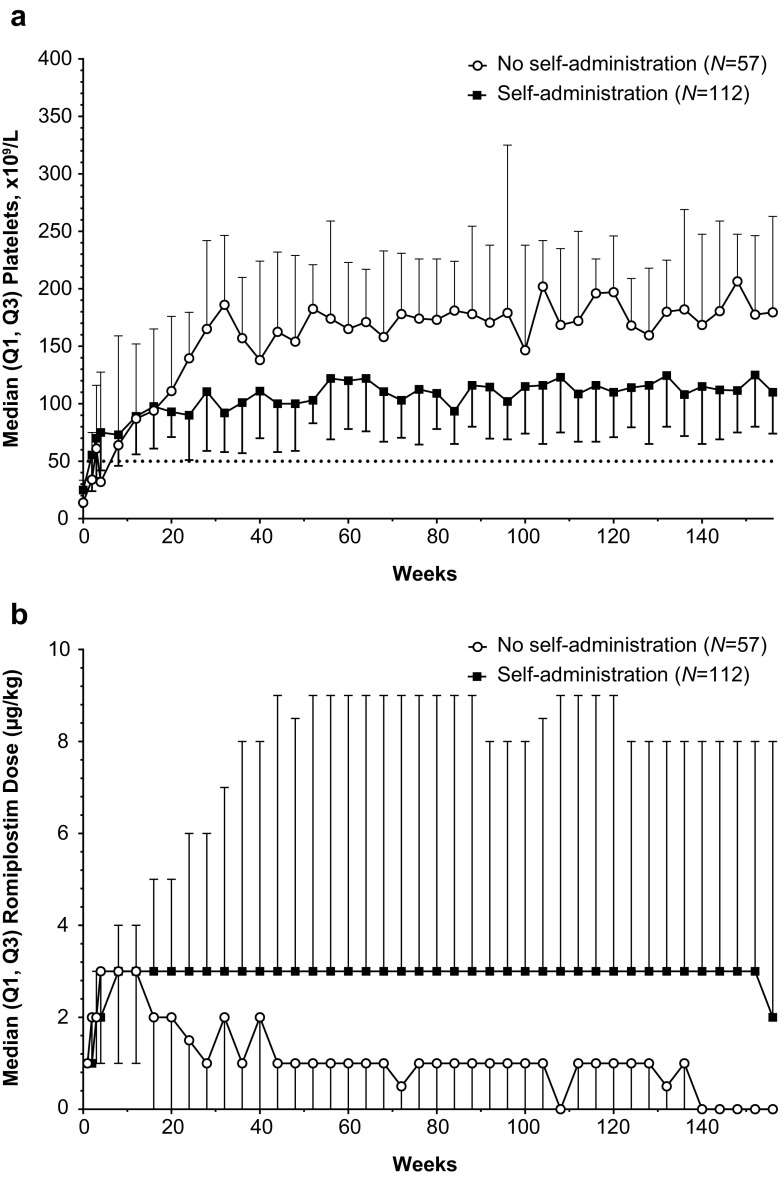
Platelet count and dose over time for patients with ITP treated with romiplostim by self-administration status. **a** Platelet count over time by self-administration status. **b** Romiplostim dose over time by self-administration status. *ITP* immune thrombocytopenia, *Q1* quartile 1, *Q3* quartile 3

Patients self-administering romiplostim were generally similar to those who did not self-administer romiplostim (Online Resource Table S7); however, there could have been either self-selection or selection by the investigators as to who self-administered romiplostim. After initiating self-administration, 51 patients (46 %) had ≥3 dose adjustments. Platelet counts and dose over time are shown for those who self-administered romiplostim (*n* = 112) and those who did not self-administer romiplostim (*n* = 57) (Fig. 4). Platelet response was observed in 98 % (110/112) of those self-administering romiplostim and 79 % (45/57) of those not self-administering romiplostim. There were no new safety concerns with patients who self-administered romiplostim.

## Discussion

Increases in bone marrow reticulin and/or collagen may be associated with benign or malignant conditions [3]. Such increases have been observed in healthy individuals [12, 21] and in patients with ITP not being treated with TPO receptor agonists [13]. In this study, an increase in modified Bauermeister score of ≥2 grades and/or collagen occurred in nine of 131 patients (6.9 %) treated with romiplostim; seven had increased reticulin only and two also had collagen. Of these nine patients, only three discontinued romiplostim early; the reasons were (1) non-response, (2) non-Hodgkin lymphoma work-up, which was negative, and (3) need for alternative therapy. While five of these nine patients had a platelet response ≤5 % of the time, two of the five received romiplostim for all 3 years per investigator’s decision. The low incidence of the presence of collagen and increased reticulin is consistent with results of past romiplostim trials; however, bone marrow biopsies were not required in those trials and were performed at the investigators’ discretion [18, 19]. This study is unique in that it prospectively examined bone marrow changes in patients with ITP treated with romiplostim.

These results are also consistent with previous studies examining the effects of TPO receptor agonists (primarily romiplostim and eltrombopag) on the bone marrow of patients with ITP [16, 17, 22–24]. In a small study of eight patients with ITP treated with TPO receptor agonists, increases of one grade on the European Consensus myelofibrosis (MF) scale were observed in four patients, with a mean duration of exposure to TPO receptor agonists of 2.7 years [23]. The MF scale has four categories (MF-0: scattered reticulin to MF-3: coarse collagen bundles) as compared with the five grades of the modified Bauermeister scale used in this study (0: no reticulin to 4: coarse fibers with collagen). A recent study of 66 patients with ITP treated with TPO receptor agonists (about half were treated with romiplostim and half with eltrombopag; some patients received both and/or experimental TPO receptor agonists) for at least 2 years found significantly higher rates of MF-2/3 associated with treatment, increasing from 9 % at the beginning of treatment to 32 % at the end of treatment [22].

For eltrombopag specifically, a study of 91 patients treated with eltrombopag found increases of one grade in 35 patients and two to three grades in two patients at 1 year; collagen was found in three patients [16]. Further, in an extension study, of 232 on-treatment bone marrow biopsies from 117 patients treated with eltrombopag for up to 5.5 years, reticulin fibrosis characterized as moderate-to-marked was found in two patients; after eltrombopag discontinuation, this fibrosis was reversible in one patient. Of note, none of the biopsies on treatment were prompted by abnormal laboratory results or symptoms related to possible bone marrow pathology [24].

The incidence of collagen and reticulin in our study increased from cohort 1 to cohort 2 to cohort 3. Incidences were too small to derive statistical significance, and the study design (biopsies on three different cohorts with the exact time of onset of bone marrow changes unknown) precludes the assessment of whether the risks of collagen and reticulin increased over time. In any case, when biopsy results were assessed by exposure at time of biopsy rather than by cohort (as bone marrow biopsies were performed earlier than scheduled in some patients), bone marrow changes did not appear to increase with time. For patients who had collagen or increases of ≥2 grades on the modified Bauermeister scale and follow-up biopsies (three of the nine), follow-up biopsies showed decreases to grade 1 or 2; therefore, bone marrow changes appear to be reversible after discontinuation of romiplostim, which is consistent with previous studies [18]. Of the nine patients with bone marrow changes, six received high doses of romiplostim (9 or 10 μg/kg); per protocol, if a patient did not respond to treatment, then the romiplostim dose was escalated up to a maximum of 10 μg/kg. Further, five of the nine patients had a low (0–5 %) percentage of time with a platelet response and three of these patients received high doses of romiplostim. A post hoc analysis of patients from cohort 3 indicated that the likelihood of bone marrow changes was lower in patients who had a lower cumulative romiplostim dose (based on unadjusted *p* value, as in Table 4). However, this finding should be interpreted with caution, as it may be that a higher cumulative romiplostim dose and the occurrence of bone marrow changes were both downstream effects of other patient characteristics such as baseline bone marrow function, i.e., these findings could be associated with each other but not causally related. Further study would be needed to determine to what extent and how romiplostim dose/exposure and bone marrow findings are related. Three of the nine patients with bone marrow changes had cytopenias: a case of neutropenia detected by laboratory results and two AEs of anemia, neither of which was considered serious or treatment-related. In one case of anemia, the patient had intraoperative bleeding from a splenectomy, and in the other, the patient had gastrointestinal bleeding. Regarding thromboembolic AEs, while 21 AEs in 15 patients (or 9 %) might seem high, it should be kept in mind that this was over the course of 169 patients receiving romiplostim for up to 3 years. The duration-adjusted rate of thromboembolic AEs, 3.9 per 100 patient-years, is similar to that seen in an integrated database of romiplostim ITP studies (7.5 per 100 patient-years) [19].

**Table 4:** Analysis of patients with and without changes in bone marrow biopsies per the modified Bauermeister scale

Cohort 3		Patients with ∆ reticulin and/or collagen^a^ (*N* = 7)	Patients with no ∆ collagen or reticulin (*N* = 51)
Age, median (Q1, Q3), years		47 (30, 60)	44 (33, 61)
ITP duration, median (Q1, Q3), years		4.1 (1.2, 9.1)	2.2 (1.2, 7.1)
Prior splenectomy rate, *n* (%)		2 (29)	17 (33)
Platelet response^b^, median (Q1, Q3)	Time to first platelet response, weeks	15 (7, 21)^c^	3 (2, 8)^d^
	Weeks with platelet response, %	5 (0, 28)	38 (28, 76)
Exposure^e^, median (Q1, Q3)	Average weekly dose, μg/kg	9 (7, 9)	2 (1, 5)
	Maximum weekly dose, μg/kg	10 (9, 10)	4 (3, 10)
	Cumulative dose, μg/kg	1207 (204, 1358)	345 (136, 610)

*ITP* immune thrombocytopenia, *Q1* quartile 1, *Q3* quartile 3, *∆* change, i.e., increase of two or more grades

^a^Five had increased reticulin only and two also had collagen

^b^Calculated only for those patients who had a platelet response within each group of patients

^c^ *N* = 4

^d^ *N* = 50

^e^Exposure was up to time of biopsy; per protocol, maximum dose was 10 μg/kg

Limitations of this study include the lack of within-patient comparison over time, as each patient was to have two biopsies only: one baseline biopsy and one after 1, 2, or 3 years of romiplostim (as opposed to having biopsies performed at all four timepoints). This study design was chosen to minimize patient discontinuation due to repeated bone marrow biopsies. This study also did not include a placebo control group, which limits analysis of the potential effects of romiplostim on the bone marrow as opposed to effects due to the progression of severe ITP. Also, the cohorts were not randomized, but enrolled sequentially, so as to ensure adequate enrollment in each of the cohorts before starting to enroll in the next cohort. While the patients in cohort 3 appear to be somewhat younger and with shorter duration of ITP, these changes are not statistically different. It is possible that patients with earlier-stage ITP were more willing to enroll in cohort 3, as there was the opportunity to receive up to 3 years of romiplostim prior to follow-up biopsy. Of note, in Ghanima et al. [22], a higher grade of fibrosis was associated with older age (*p* = 0.01), which we did not find in this study; the association with ITP duration was not significant (*p* ≥ 0.05 in both analyses) in either study. A further limitation is that while all patients had bone marrow biopsies done at baseline, some patients did not have follow-up biopsies (32/169, or 19 %) and a few did not have evaluable biopsies. The rate of bone marrow changes of collagen and/or ≥2 grades on the modified Bauermeister scale was low and limited statistical interpretation and comparison, particularly for any subgroups, and there was a relatively short duration of follow-up in determining the clinical importance of bone marrow changes. Lastly, the exact time of when bone marrow changes occurred is not known; it can only be stated that it occurred prior to the time of biopsy, which limits assessment of any relationship between time of exposure and bone marrow changes.

In conclusion, our data indicate that in patients with ITP treated with romiplostim, there was a low incidence of bone marrow changes (nine in 131 evaluable biopsies, or 6.9 %). Whether these observations are specifically related to romiplostim exposure and response, i.e., those patients responding well at lower doses being at lower risk of developing bone marrow changes than those requiring higher doses, requires further evaluation. The low incidence of both collagen and increased reticulin is consistent with previous romiplostim studies.

Given the caveats described above, although analyses of romiplostim ITP trials of over 600 patients treated for up to 5 years have not shown a bone marrow signal [8, 19], it is possible that longer duration of treatment and higher doses of TPO receptor agonists may lead to an increase in bone marrow changes, which in turn could lead to changes in peripheral blood counts. As physicians may be aware, as romiplostim may increase the risk of reticulin fibers in the bone marrow, the prescribing information recommends that detection of any peripheral blood cell abnormalities (such as tear drop cells or any other phenomena leading to clinical suspicion) may necessitate a bone marrow biopsy [25]. If an increase in reticulin or fibrosis is found in the bone marrow, physicians may wish to discontinue treatment with TPO receptor agonists. With regard to a potential increase in reticulin/collagen deposition during treatment with TPO receptor agonists, there is not yet consensus on optimal follow-up or whether and at what intervals bone marrow biopsies should be performed in patients treated with TPO receptor agonists. In cases of patients having cytopenias, performing bone marrow biopsies seems advisable.

## Electronic supplementary material

Below is the link to the electronic supplementary material.

ESM 1 (DOCX 41 kb)
