# Supplementary material for: Changes in bone marrow morphology in adults receiving romiplostim for the treatment of thrombocytopenia associated with primary immune thrombocytopenia
Source: Ann Hematol. 2016 Apr 30;95:1077–87. doi: 10.1007/s00277-016-2682-2 (PMC4889627; doi:10.1007/s00277-016-2682-2)
Supplement: Supplementary file 1 — (DOCX 41 kb) [file 277_2016_2682_MOESM1_ESM.docx]

**Electronic Supplementary Information**

**Title:** Changes in bone marrow morphology in adults receiving romiplostim for the treatment of thrombocytopenia associated with primary immune thrombocytopenia

**Authors:** Ann Janssens^1^ · Francesco Rodeghiero^2^ · David Anderson^3^ · Beng H. Chong^4^ · Zoltán Boda^5^ · Ingrid Pabinger^6^ · Libor Červinek^7^ · Deirdra R. Terrell^8^ · Xuena Wang^9^ · Janet Franklin^9^

**Affiliations:** ^1^ University Hospitals Leuven, Leuven, Belgium

^2^ San Bortolo Hospital, Vicenza, Italy

^3^ Dalhousie University, Halifax, NS, Canada

^4^ St. George Hospital, Sydney, Australia

^5^ University of Debrecen, Clinical Center Department of Medicine, Thrombosis Haemostasis Center, Debrecen, Hungary

^6^ Universitätsklinik für Innere Medizin I, Medizinische Universität Wien, Vienna, Austria

^7^ University Hospital Masaryk University, Brno, Czech Republic

^8^ University of Oklahoma Health Sciences Center, Oklahoma City, OK, USA

^9^ Amgen Inc., Thousand Oaks, CA, USA

**Correspondence:** Ann Janssens

Email: ann.janssens@uzleuven.be

Online Resource Table S1. Modified Bauermeister scale

| Grade | Quantification of bone marrow reticulin and collagen |
| --- | --- |
| 0 | No reticulin |
| 1 | Occasional fine individual fibers and foci of a fine fiber network |
| 2 | Fine fiber network throughout most of the section; no coarse fibers |
| 3 | Diffuse fiber network with scattered thick coarse fibers but no mature collagen (negative trichrome staining) |
| 4 | Diffuse, coarse fiber network with areas of collagenization  (positive trichrome staining) |

Online Resource Table S2. Patient disposition for the 3-year study

| Characteristic, *n* (%) | Cohort 1 (*N*=50) | Cohort 2 (*N*=50) | Cohort 3 (*N*=69) | Total (*N*=169) |
| --- | --- | --- | --- | --- |
| Completed 3-year study | 23 (46) | 33 (66) | 47 (68) | 103 (61) |
| Early discontinuation of 3-year study | 27 (54) | 17 (34) | 22 (32) | 66 (39) |
| Withdrew consent | 8 (16) | 9 (18) | 6 (9) | 23 (14) |
| No response | 5 (10) | 2 (4) | 3 (4) | 10 (6) |
| Death | 4 (8) | 2 (4) | 1 (1) | 7 (4) |
| Adverse event | 3 (6) | 1 (2) | 2 (3) | 6 (4) |
| Required alternative therapy | 1 (2) | 2 (4) | 3 (4) | 6 (4) |
| Administrative decision | 2 (4) | 0 | 3 (4) | 5 (3) |
| Ineligibility determined | 0 | 0 | 1 (1) | 1 (1) |
| Noncompliance | 0 | 0 | 1 (1) | 1 (1) |
| Lost to follow-up | 1 (2) | 0 | 0 | 1 (1) |
| Pregnancy | 0 | 1 (2) | 1 (1) | 2 (1) |
| Other^a^ | 3 (6) | 0 | 2 (3) | 4 (2) |
| Patients with bone marrow biopsies after receiving romiplostim^b^ | 39 (78) | 40 (80) | 58 (84) | 137 (81) |

^a^ Other includes transportation issues, elevated platelet count, per investigator (Cohort 1), changed physician, and positive antibody (Cohort 3)
^b^ Three patients in Cohort 1, three in Cohort 2, and 10 in Cohort 3 had bone marrow biopsies at end of treatment because of early discontinuation

Online Resource Table S3 Grade Changes in Modified Bauermeister Grading Scale (N = 169)

| Baseline Grade^a^ | Worst Post-Baseline Grade | | | | | Unevaluable  n (%) | Missing  n (%) | Total  n (%) |
| --- | --- | --- | --- | --- | --- | --- | --- | --- |
|  | 0  n (%) | 1  n (%) | 2  n (%) | 3  n (%) | 4  n (%) |  |  |  |
| 0 | 8 (4.7) | 24 (14.2)^c^ | 3 (1.8)^d^ | 0 (0) | 0 (0) | 0 (0) | 8 (4.7) | 43 (25.4) |
| 1 | 8 (4.7)^b^ | 67 (39.6) | 10 (5.9)^c^ | 4 (2.4)^d^ | 2 (1.2)^d^ | 6 (3.6) | 22 (13.0) | 119 (70.4) |
| 2 | 0 (0) | 2 (1.2) | 2 (1.2) | 1 (0.6) | 0 (0) | 0 (0) | 2 (1.2) | 7 (4.1) |
| Total | 16 (9.5) | 93 (55.0) | 15 (8.9) | 5 (3.0) | 2 (1.2) | 6 (3.6) | 32 (18.9) | 169 (100) |

All available on-study bone marrow biopsies, except follow-up biopsies performed at 12 weeks after the discontinuation of romiplostim at end-of- study visits, are included in the analysis.

^a^ There were no patients with grade 3 or grade 4 biopsies at baseline. Per protocol, the maximum allowable baseline grade was 3.

^b^ Reduced by 1 severity grade on the modified Bauermeister scale.

^c^ Increased by 1 severity grade on the modified Bauermeister scale.

^d^ Increased modified Bauermeister grade: an increase by ≥ 2 severity grades or an increase to grade 4 on the

modified Bauermeister scale.

Online Resource Table S4. Overall safety for each cohort in the 3-year period

| AE, patient incidence *n* (%) | Cohort 1 (*N*=50) | Cohort 2 (*N*=50) | Cohort 3 (*N*=69) | Total (*N*=169) |
| --- | --- | --- | --- | --- |
| Any AE | 46 (92) | 45 (90) | 67 (97) | 158 (94) |
| Treatment-related AE | 14 (28) | 22 (44) | 24 (35) | 60 (36) |
| Any serious AE | 16 (32) | 12 (24) | 28 (41) | 56 (33) |
| Treatment-related serious AE | 1 (2) | 2 (4) | 3 (4) | 6 (4) |
| Thromboembolic AE | 7 (14) | 3 (6) | 5 (7) | 15 (9) |
| Withdrawal from study due to AE | 6 (12)^a^ | 2 (4)^b^ | 3 (4)^c^ | 11 (7)^d^ |
| Death | 4 (8) | 2 (4) | 1 (1) | 7 (4) |

*AE* adverse event

^a^ Arthralgia, cardiac disorder, cerebral hemorrhage, fungal sepsis, portal vein thrombosis, pulmonary hemorrhage

^b^ Abnormal lymphocyte morphology, acute renal failure

^c^ Lichenoid keratosis, thrombosis, venous thrombosis in the limb

^d^ Of these, arthralgia, lichenoid keratosis, and venous thrombosis in the limb were attributed to romiplostim

Online Resource Table S5. Fatalities

| Cohort | Description |
| --- | --- |
| 1 | 1. Subdural hematoma after the patient fell and sustained a head injury; platelet count was 112×10^9^/L a month earlier and 61×10^9^/L the day of the head injury  2. Fungal sepsis in a patient with longstanding corticosteroid use, including through the first month on romiplostim; corticosteroids were not used in the 6 months prior to the development of sepsis  3. Cerebral hemorrhage; 4 days prior, platelet count was 8×10^9^/L  4. Pulmonary hemorrhage; 1 day before death, platelet count was 60×10^9^/L. Death occurred 18 days after the patient had a pulmonary thrombosis, which was treated with the anticoagulant acenocoumarol |
| 2 | 1. Acute renal failure; patient had a history of chronic renal insufficiency  2. Suicide |
| 3 | 1. Multiple thromboses/multiorgan failure of the heart, kidneys, and lungs |

Online Resource Table S6. AEs by reticulin status

| AEs, *n* (%) | Patients with ∆ reticulin/collagen (*N*=9) | Patients with no ∆ reticulin/collagen (*N*=122) |
| --- | --- | --- |
| All AEs | 9 (100.0) | 117 (95.9) |
| Grade ≥2 | 6 (66.7) | 101 (82.8) |
| Grade ≥3 | 5 (55.6) | 58 (47.5) |
| Grade ≥4 | 3 (33.3) | 20 (16.4) |
| Serious AEs | 4 (44.4) | 37 (30.3) |
| Leading to discontinuation of investigational product | 1 (11.1) | 6 (4.9) |
| Leading to discontinuation from study | 1 (11.1) | 2 (1.6) |
| Fatal AEs | 0 | 2 (1.6)^a^ |
| Treatment-related AEs | 2 (22.2) | 44 (36.1) |
| Grade ≥2 | 1 (11.1) | 22 (18.0) |
| Grade ≥3 | 1 (11.1) | 9 (7.4) |
| Grade ≥4 | 0 | 1 (0.8) |
| Serious AEs | 1 (11.1) | 3 (2.5) |
| Leading to discontinuation of investigational product | 0 | 0 |
| Leading to discontinuation from study | 0 | 0 |
| Fatal AEs | 0 | 0 |

*AE* adverse event, *∆* change i.e., increase of two or more grades

^a^ While there were a total of seven deaths on study, five patients did not have reticulin grades, as they died prior to planned biopsy time and thus are not included in this table

Online Resource Table S7. Baseline characteristics by self-administration status

| Characteristic | Self-administration  (*N*=112) | No self-administration (*N*=57) |
| --- | --- | --- |
| Female, *n* (%) | 79 (71) | 35 (61) |
| Age, mean (SD), years | 50 (17) | 50 (18) |
| Platelet count, median (range), ×10^9^/L | 25 (1–93) | 14 (1–130) |
| Hemoglobin, median (range), g/L | 137 (87–168) | 141 (94–168) |
| Absolute neutrophil count, median (range), ×10^9^/L | 4.9 (1.2–21.3) | 5.6 (1.1–15.9) |

*SD* standard deviation
